# The 25 kDa Subunit of Cleavage Factor Im Is a RNA-Binding Protein That Interacts with the Poly(A) Polymerase in *Entamoeba histolytica*

**DOI:** 10.1371/journal.pone.0067977

**Published:** 2013-06-28

**Authors:** Marisol Pezet-Valdez, Jorge Fernández-Retana, Juan David Ospina-Villa, María Esther Ramírez-Moreno, Esther Orozco, Socorro Charcas-López, Jacqueline Soto-Sánchez, Guillermo Mendoza-Hernández, Mavil López-Casamicha, César López-Camarillo, Laurence A. Marchat

**Affiliations:** 1 Programa Institucional de Biomedicina Molecular, Escuela Nacional de Medicina y Homeopatía del IPN, Guillermo Massieu Helguera #239, Fracc. La Escalera, Ticomán, México D.F., Mexico; 2 Doctorado en Biotecnología en Red, Escuela Nacional de Medicina y Homeopatía del IPN, Guillermo Massieu Helguera #239, Fracc. La Escalera, Ticomán, México D.F., Mexico; 3 Departamento de Infectómica y Patogénesis Molecular, Centro de Investigación y de Estudios Avanzados, Instituto Politécnico Nacional, México D.F., Mexico; 4 Departamento de Bioquímica, Facultad de Medicina, Universidad Nacional Autónoma de México, México D.F., Mexico; 5 Posgrado en Ciencias Genómicas, Universidad Autónoma de la Ciudad de México, México D.F., Mexico; University of Ottawa, Canada

## Abstract

In eukaryotes, polyadenylation of pre-mRNA 3´ end is essential for mRNA export, stability and translation. Taking advantage of the knowledge of genomic sequences of *Entamoeba histolytica*, the protozoan responsible for human amoebiasis, we previously reported the putative polyadenylation machinery of this parasite. Here, we focused on the predicted protein that has the molecular features of the 25 kDa subunit of the Cleavage Factor Im (CFIm25) from other organisms, including the Nudix (nucleoside diphosphate linked to another moiety X) domain, as well as the RNA binding domain and the PAP/PAB interacting region. The recombinant EhCFIm25 protein (rEhCFIm25) was expressed in bacteria and used to generate specific antibodies in rabbit. Subcellular localization assays showed the presence of the endogenous protein in nuclear and cytoplasmic fractions. In RNA electrophoretic mobility shift assays, rEhCFIm25 was able to form specific RNA-protein complexes with the *EhPgp5* mRNA 3´ UTR used as probe. In addition, Pull-Down and LC/ESI-MS/MS tandem mass spectrometry assays evidenced that the putative EhCFIm25 was able to interact with the poly(A) polymerase (EhPAP) that is responsible for the synthesis of the poly(A) tail in other eukaryotic cells. By Far-Western experiments, we confirmed the interaction between the putative EhCFIm25 and EhPAP in *E. histolytica*. Taken altogether, our results showed that the putative EhCFIm25 is a conserved RNA binding protein that interacts with the poly(A) polymerase, another member of the pre-mRNA 3´ end processing machinery in this protozoan parasite.

## Introduction

RNA processing is an essential event for gene expression regulation. Particularly, the poly(A) at the 3´ end of most eukaryotic mRNA influences mRNA stability, translation, and transport [1-3]. Additionally, interactions between the different molecular machineries participating in mRNA synthesis, transcription termination, mRNA 3´ end formation, 5´ end capping, splicing and translation, revealed a functional link between all mRNA processing events [4-9]. Polyadenylation is a two-step nuclear reaction that involves pre-mRNA 3´ end cleavage at the cleavage and polyadenylation site (poly(A) site) followed by the addition of a polyadenine tail [10-13]. Both reactions depend on trans-acting factors interacting in a coordinated way with specific motifs in pre-mRNA 3´ untranslated region (UTR). In Mammals, the poly(A) site generally denoted by the CA dinucleotide [14] is flanked by the hexameric A(A/U) UAAA polyadenylation signal (PAS) and the GU/U-rich downstream element (DSE). Additional elements include U-rich (UUUU, UGUA or UAUA) and G-rich upstream elements, as well as the G-rich downstream element [15,16]. The 160 kDa subunit of the cleavage and polyadenylation specificity factor (CPSF) recognizes the PAS [17], while the N-terminal RNA recognition motif (RRM) from the 64 kDa subunit of the cleavage stimulatory factor (CstF) binds the DSE [18]. Cleavage factor I (CFIm) interacts with the UGUAN (N=A>U≥C/G) motif [19,20], which promotes the recruitment of CFIIm, poly(A) polymerase (PAP), poly(A) binding protein (PABP), transcriptional coactivator PC4, symplekin and carboxyterminal domain of the RNA polymerase II largest subunit (RNA pol II CTD) [21]. CPSF-73 is thought to perform pre-mRNA cleavage [22], before PAP catalyzes the poly(A) tail synthesis [21].

Initial reports suggested that active CFIm is an heterodimer formed by the small 25 kDa subunit interacting with one of the larger 59 or 68 kDa subunits in Human [23,24]. CFIm25 belongs to the Nudix hydrolase superfamily, whereas the structurally related CFIm59 and CFIm68 contain a RRM [23,25]. Several studies suggested that the 25 kDa subunit is an essential component for the normal physiology of the cell [26]. Later, crystallographic analyses revealed that CFIm25 interacts with the UGUAN motif as a homodimer constituted by two deeply interweaved subunits [27-29]. A recent biochemical study evidenced that CFIm forms a stable heterotetramer through the dimerization of CFIm25 and binding to either the 59 or 68 kDa subunit [30]. Together with CPSF and CstF, the binding of CFIm to UGUAN sequence contributes to the selection of both canonical and non-canonical poly(A) sites [19,23]. Knockdown of CFIm25 or CFIm68 caused an upstream shift in poly(A) site selection for both *TIMP2* and *DHFR* genes that have alternative poly(A) sites in their 3´ UTR, which is consistent with the idea that both 25 kDa and 68 kDa subunits are essential components of CFIm. On the other hand, CFIm59 functions similarly to CFIm25 and CFIm68 in stimulating the utilization of downstream poly(A) sites [30,31]. Additionally, CFIm25 stabilizes the interaction of CPSF with PAS, increases the cleavage rate *in vitro* and interacts with PAP and PABP [24,32]. Moreover, it is able to bind the splicing factor U2AF65, establishing a functional link between the different molecular events of mRNA processing [33].

*Entamoeba histolytica*, the protozoan parasite causative of human amoebiasis, is responsible for intestinal dysentery and hepatic abscesses that result in 70,000-100,000 deaths a year, making it a leading cause of parasitic death in humans [34]. Taking advantage of the genomic information obtained from the *E. histolytica* genome sequence project and the conservation of proteins through evolutionary scale, we have recently identified the cleavage and polyadenylation machinery [35] and initiated the characterization of EhPAP [36] and EhPC4 (Hernandez-de la Cruz et al., in preparation) in this human pathogen. Notably, we identified a gene for the putative 25 kDa subunit of the Cleavage Factor Im, but we did not find any genes corresponding to the higher molecular weight subunits found in human [35]. In this paper, we focused on the study of the putative EhCFIm25 from *E. histolytica*. Our results evidenced that it is a conserved RNA binding protein that interacts with the poly(A) polymerase, another member of the pre-mRNA 3´ end processing machinery of this protozoan parasite.

## Materials and Methods

### Ethics statements

This study was carried out in strict accordance with the recommendations of the Guide for the Use of Laboratory Animals of the Investigation Center and Advanced Studies of the National Polytechnic Institute. The protocols and experiments were approved by the Institutional Animal Care of the Investigation Center and Advanced Studies of the National Polytechnic Institute. Animals were kept in environmentally controlled animal facilities at the Investigation Center and Advanced Studies of the National Polytechnic Institute. All surgery was performed under sodium pentobarbital anesthesia and efforts were always made to minimize suffering.

### *In silico* analysis of EhCFIm25 protein sequence

The predicted amino acid (aa) sequence of the putative EhCFIm25 (C4M2T1) previously reported by us [35] was used to determine identity/similarity percentages and e values to related proteins by BLAST (http://www.expasy.org/tools/blast/). Homologous protein sequences from diverse organisms were aligned by ClustalW software (http://www.ch.embnet.org/software/ClustalW.html/), allowing gap penalties of 10 to maximize protein homology. Structural domains and sequence patterns were predicted by Motif Scan (http://myhits.isb-sib.ch/cgi-bin/motif_scan) and Scan Prosite (http://www.expasy.org/tools/scanprosite/) programs.

For phylogenetic studies, the full-length amino acid sequence of the 25 kDa subunit of Cleavage Factor I from various organisms were aligned by ClustalW and used to build the corresponding tree by the Neighbor-Joining method [37] through the MEGA (Molecular Evolutionary Genetics Analysis) software version 5.05 [38]. The bootstrap consensus rooted tree inferred from 1000 replicates was taken to represent the evolutionary history of the analyzed proteins. Branches corresponding to partitions reproduced in less than 50% bootstrap replicates are collapsed. The percentage of replicate trees in which the associated proteins clustered together in the bootstrap test (1000 replicates) is shown next to the branches. The evolutionary distances were computed using the number of differences method [39] and are in the units of the number of amino acid differences per sequence. All positions containing gaps and missing data were eliminated.

The secondary structure of the putative EhCFIm25 was analyzed through Chou & Fasman Secondary Structure Prediction (CFSSP) server at http://www.biogem.org/tool/chou-fasman/. The three-dimensional structure was predicted using the crystal data of *Homo sapiens* CFIm25 with the Swiss-Model software (http://www.expasy.ch/swissmod/), and visualized through PyMol (http://pymol.sourceforge.net/) program. Model was validated by Ramachandran graph and RMSD value.

### *E. histolytica* culture

Trophozoites of clone A (strain HM1-IMSS) were axenically grown in TYI-S-33 medium at 37°C [40] and harvested in logarithmic growth phase for all experiments.

### Cloning and sequencing of the *EhCFIm25* gene

The full-length *EhCFIm25* sequence (768 nt) reported at locus EHI_077110 in the Amoeba database (http://amoebadb.org/amoeba/) was PCR amplified from genomic DNA of clone A trophozoites using sense EhCFIm25-S (5´-CCCCG G A T C CAATAAA**ATG**GAAGAGCA-3´) and antisense EhCFIm25-AS (5´-CCCCCG A A T T CTTAACCATAAATCATAAGATACC-3´) specific primers with high fidelity Taq DNA polymerase (2.0 U) (Invitrogen). *Bam*HI and *Eco*RI restriction sites are underlined, the translation initiation site is in bold. Amplification was performed as follows: 94°C for 5 min and 30 cycles at 94°C for 35 s, 66°C for 50 s and 72°C for 1 min, plus a final extension step at 72°C for 7 min. The PCR product was purified and cloned in frame into the pRSET A expression vector (Invitrogen) to generate the recombinant pRSET-*EhCFIm25* plasmid. The construct was confirmed by automated DNA sequencing in an ABI-PRISM 310 sequencer (Applied Biosystem).

### Expression and purification of recombinant EhCFIm25 (rEhCFIm25) protein

The pRSET-*EhCFIm25* plasmid was used to transform competent *E. coli* BL21 (DE3) pLysS bacteria grown at 37°C in 2-TY medium containing 100 μg/ml ampicillin and 34 μg/ml chloramphenicol. The recombinant polypeptide (rEhCFIm25) was expressed as a 6x-His-labeled fusion protein after induction with 1 mM isopropyl beta-D-thiogalactopyranoside (IPTG) at 37°C for 3 h. Cells were harvested by centrifugation at 14,000 rpm for 5 minutes, resuspended in lysis buffer (50 mM NaH_2_PO_4_, 300 mM NaCl, 10 mM imidazole, pH 8.0) and lysed by sonication at 4°C. After centrifugation at 14,000 rpm for 5 minutes, the supernatant corresponding to total protein extracts was collected and used to purify near to homogeneity the recombinant EhCFIm25 through Ni^2+^-NTA affinity chromatography under non denaturing conditions according to the manufacturer recommendations (Qiagen). The identity and integrity of rEhCFIm25 were confirmed by 10% SDS-PAGE and Western blot assays using anti-6x-His tag antibodies (Roche) at 1:10000 dilution and the ECL Plus Western blotting detection system (Amersham).

### Production of polyclonal antibodies against EhCFIm25

Purified rEhCFIm25 was subjected to preparative 10% SDS-PAGE, electroeluted from Coomassie stained-gels and subsequently used as antigen to immunize a New Zeland male rabbit. An initial dose of 200 μg in 90 μl complete Freund’s adjuvant (Sigma) was subcutaneously inoculated into the animal, and then three doses of 200 μg in 90 μl incomplete Freund’s adjuvant were injected every seven days. One week after the last immunization, the rabbit was bled and polyclonal antiserum was obtained. IgGs were purified through protein G sepharose 4 Fast Flow (Amersham Biosciences) chromatography and tested for reactivity against rEhCFIm25 protein by Western blot assays (dilution 1:1000).

### Western blot assays

Cytoplasmic (CE) and nuclear (NE) extracts from clone A trophozoites were prepared as described [41] with some modifications. Briefly, 10^7^ trophozoites were harvested, washed twice with cold PBS pH 6.8, resuspended in four volumes of Buffer A (10 mM HEPES, pH 7.9, 1.5 mM MgCl_2_, 10 mM KCl, 0.5 mM DTT, 0.5 mM PMSF) supplemented with protease inhibitors (0.5 mM PMSF; 2 mM benzamidine; 5 μg/ml of each aprotinin, pepstatin A, leupeptin, and E-64) and incubated for 20 min at 4 °C, monitoring nuclei integrity by phase-contrast microscopy. After centrifugation at 14,000 rpm for 1 min, the supernatant corresponding to CE was collected and stored at -70 °C until being used. Nuclei contained in the pellet were lysed by incubation for 40 min at 4 °C in 50 μl Buffer C (20 mM HEPES, pH 7.9, 0.42 mM NaCl, 1 mM EDTA, 1mM PMSF, 1 mM EGTA, 0.5 mM DTT) in the presence of protease inhibitors. After centrifugation at 14,000 rpm for 5 min at 4 °C, the supernatant corresponding to NE was collected and stored at -70 °C until being used. CE and NE were subjected to 10% SDS-PAGE and electrotransferred to a nitrocellulose membrane that was incubated with anti-EhCFIm25 polyclonal antibodies (1:100 dilution) and goat anti-rabbit IgG horseradish peroxidase secondary antibody (Zymed) (1:10000 dilution). Then, proteins were revealed with the ECL Plus Western blotting system (Amersham). As controls, we used anti-EhPC4 antibodies that specifically identified the putative *E. histolytica* transcription and polyadenylation factor EhPC4 in the nuclear fraction [42] and antibodies against EhPAP that has been previously located in both CE and NE [36]. In some experiments, proteins were analyzed in the presence of a higher concentration of β-mercaptoethanol (8%) and 8 M urea, in order to improve reducing conditions.

### RT-PCR assays

Total RNA from clone A trophozoites was obtained by Trizol (Invitrogen). cDNA were synthesized using 1 μg total RNA, 100 ng oligo (dT18), 100 mM DTT, 10 mM dNTPs, 40 U SUPERase-in (Ambion) and 200 U Superscript II reverse transcriptase (Invitrogen) in first-strand buffer, for 1 h at 42°C. Semi-quantitative PCR assays were performed with 1⁄5 volume of the reverse transcription mixture, 10 mM each dNTPs, 5 mM MgCl_2_ and 2.5 units of *Taq* DNA polymerase (Invitrogen) [42], using 5´-GAACAACAGAACCAATGCAAG-3´ (sense) and 5'-CAATTTTTAATCTTCCTCCAACA-3' (antisense) as internal primers for *EhCFIm25* transcripts. Amplification was performed as follows: 94 ºC for 5 min; 94 ºC for 1 min, 53 ºC for 1 min and 72 ºC for 1 min, for 25 cycles, and a final extension step at 72 ºC for 7 min. As an internal control, we used the *actin* (5´-AGCTGTTCTTTCATTA TATGC-3´) sense and (5´-TTCTCTTTCAGCAGTA GTGGT-3´) antisense specific primers. RT-PCR products were separated through electrophoresis in 7% polyacrylamide-TBE gel, ethidium bromide stained and observed in a Gel-Doc apparatus (Bio-Rad).

### RNA Electrophoretic Mobility Shift Assays (REMSA)

The radiolabeled RNA probe was obtained by retrotranscription of the recombinant plasmid pBS-PSIII^156^ [43] using the Riboprobe^TM^ kit (Promega) in the presence of [α-^32^P] UTP (3000 Ci/mmol). A control RNA probe without any label was similarly synthesized. Then, 5 x 10^5^ cpm PSIII^156^ and various amounts of rEhCFIm25 were incubated in binding buffer (10 mM HEPES, pH 7.9, 40 mM KCl, 1 mM DTT, 4 mM MgCl_2_, 4 mM spermidine, 5% glycerol) at 4 °C for 15 min in 20 μl of final volume. Subsequently, RNase A + T1 (10 μg + 20 U) (Sigma) were added to the mixture that was incubated at room temperature for 15 min. Then, heparin (5 mg/ml) was added, and the mixture was incubated for an additional 10 min. For competition assays, we used a 350-fold molar excess of the PSIII^156^-unlabeled probe or tRNA. In some assays, RNase or proteinase K (20 U) were added as controls. For supershift assays, the rEhCFIm25 was pre-incubated with specific rabbit polyclonal antibody at 4 ºC for 5 min, prior to the addition of the RNA probe. RNA-protein complexes were resolved at 130 V for 3 h on pre-electrophoresed 6% non-denaturing PAGE. Gels were vacuum-dried, and RNA-protein interactions were detected by scanning in a PhosphorImager apparatus and submitted to densitometry analysis.

### Pull-Down assays

Pull-Down assay was performed as described by Reyes del Valle and Del Angel [44] with some modifications using rEnCFIm25 as bait and NE as prey. The rEhCFIm25 protein (100 µg) and 1 ml Ni^2+^-NTA (Qiagen) were mixed in batch for 2 h at room temperature in non-denaturing conditions. The resulting rEhCFIm25-Ni^2+^-NTA column was then washed with 50 mM NaH_2_PO_4,_ 750 mM NaCl, 20 mM Imidazol, 1% Triton X-100, 10 mM β-mercaptoethanol, pH 8, and kept at 4°C until being used. Then, NE (100 µg) that were previously dialyzed and passed through another Ni^2+^-NTA column to eliminate non-specific proteins interaction, were subjected to the rEhCFIm25-Ni^2+^-NTA column. After interaction and extensive wash with washing buffer, proteins were eluted using 500 mM Imidazol. Eluted proteins corresponding to three independent Pull-Down assays were pooled, concentrated using the Amicon ultra 4 10K of Millipore system and subjected to 9% SDS-PAGE followed by Coomassie Brilliant Blue staining. Finally, selected bands were excised, trypsin in gel digested and subjected to LC/ESI-MS/MS tandem mass spectrometry analysis as we previously described [45]. Protein identity was confirmed through NCBI database search.

### Far-Western blot assays

Far-Western experiments were performed according to the protocol previously described by Reyes del Valle and Del Angel [44] with some modifications. The purified rEhCFIm25 protein (50 µg) was dialyzed against interaction buffer (200 mM NaCl-Tween 5%) for 4 h, subjected to 10% SDS-PAGE and electrotransferred to nitrocellulose membrane. Membrane was blocked with 5% non-fat milk in PBST buffer (PBS 1% - Tween 20 0.05%) and incubated for 4 h at room temperature in interaction buffer (200 mM NaCl - Tween 5%) containing rEhPAP protein (50 µg/ml) that was obtained as a 6x-His-labeled fusion protein [36], purified by Ni^2+^-NTA affinity chromatography (Qiagen) in non denaturing conditions and dialyzed against interaction buffer. After washing with interaction buffer, membrane was incubated with specific mouse antibodies anti-EhPAP (1:2000 dilution). Finally, goat anti-mouse IgG horseradish peroxidase secondary antibody (Zymed) (1:2000) was added and signal was developed by the chemiluminescence ECL system (Amersham). Anti-EhCFIm25 (1:3000) and anti-His (1:2000) antibodies were used as controls.

In other assays, a membrane containing the purified rEhPAP protein (50 µg) was incubated with purified rEhCFIm25 protein in the same experimental conditions and proteins were revealed using specific rabbit antibodies anti-EhCFIm25 (1:10000 dilution). Anti-EhPAP (1:2000 dilution) and anti-His (1:10000 dilution) antibodies were used as controls.

## Results

### Predicted EhCFIm25 proteins conserve the molecular characteristics of homologous proteins

**Figure 1:**
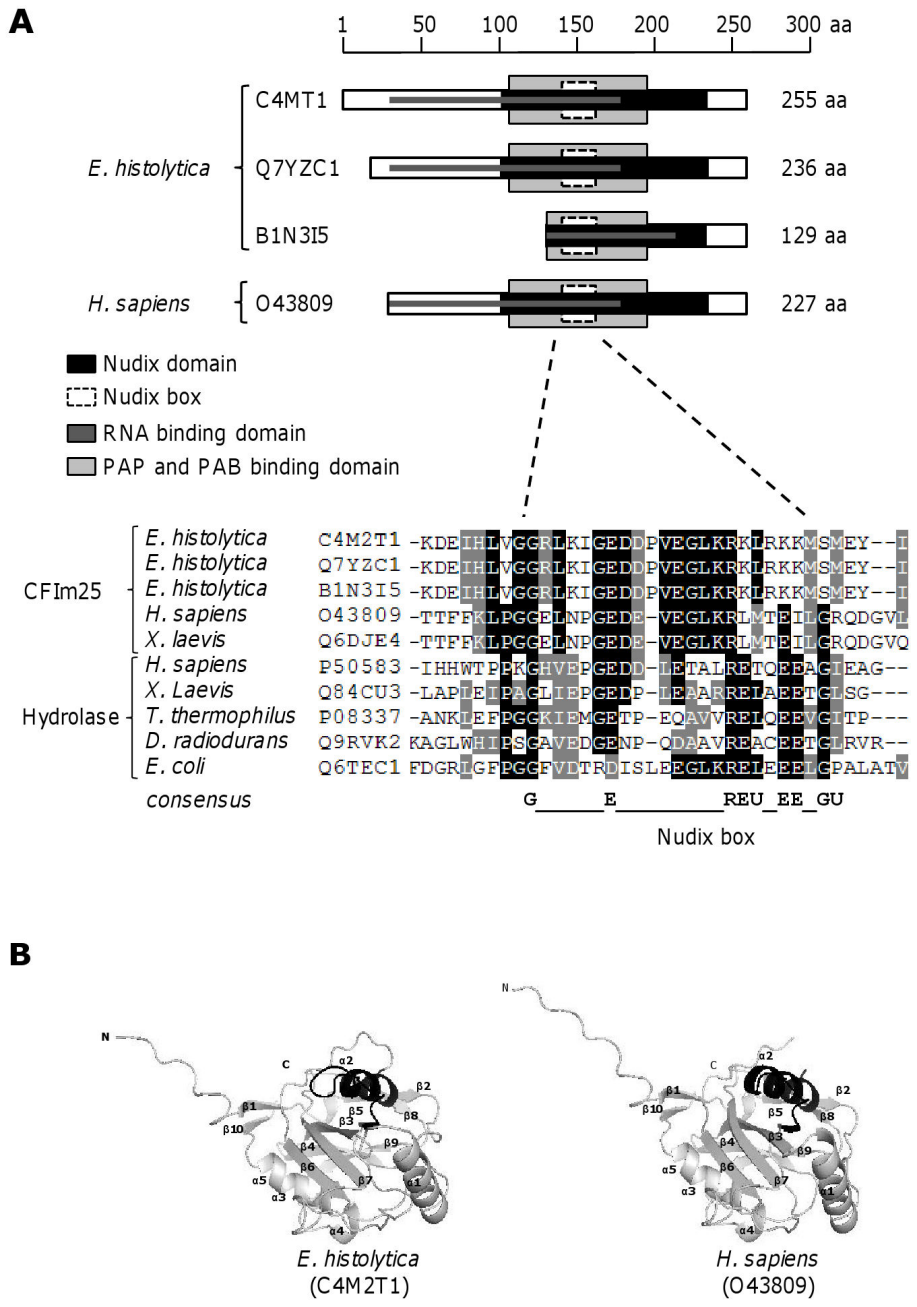
Molecular characteristics of predicted EhCFIm25. A) Comparative molecular organization of CFIm25 proteins from *E. histolytica* and human. Upper panel, Schematic representation. The scale at the top indicates the size in aa. Numbers at the right are relative to the initial methionine in each protein. Lower panel, ClustalW sequence alignment of Nudix box. Black box, identical aa; grey box, similar aa. X, any residue; U, hydrophobic residue. D) Three dimensional organization of CFIm25 proteins from *E. histolytica* (left) and human (right). 3D modeling of EhCFIm25 was obtained using crystal data from human CFIm25 as template. The Nudix box is in black color. The UniProt KnowledgeBase database accession number for each predicted protein is indicated.

Using the first available DNA sequences of *E. histolytica* genome database, we have previously reported that locus EHI_077110 is an intronless gene (768 bp) that encodes a 255 aa putative EhCFIm25 (C4M2T1) [35]. BLAST analysis of the actualized *E. histolytica* genome database confirmed the relevance of locus EHI_077110 and revealed that two adjacent intronless genes also encode putative homologues to human CFIm25: locus EHI_077000 (711 bp) is identical to locus EHI_077110 but lacks 57 bp at the 5´ end, encoding a 236 aa protein (Q7YC1); locus EHI_077220 (390 bp) encodes a 129 aa truncated polypeptide (B1N3I5) that corresponds to the amino terminus of the other proteins (Figure 1A). The three predicted proteins share 27-35% identity and 47-65% similarity with CFIm25 proteins from protozoa, nematodes, plants, vertebrate animals and human. Interestingly, results evidenced a strong homology with the unique putative CFIm25 identified in *E. dispar*, the non pathogenic *Entamoeba* , with e-values ranging from 4e-67 to e-114, and identity and similarity percentage ranging from 86 to 96%, and 89 to 98%, respectively (Table 1).

**Table 1:** Comparison of *E. histolytica* predicted proteins with CFIm25 proteins from other organisms.

		*E. histolytica* predicted protein								
		C4M2T1			Q7YZC1			B1N3I5		
Organism	Accession number^a^	e-value	I	S	e-value	I	S	e-value	I	S
*Homo sapiens*	O43809	5e-21	32	55	6e-21	32	55	5e-13	35	58
*Mus musculus*	Q9CQF3	5e-21	32	55	6e-21	32	55	5e-13	35	58
*Gallus gallus*	E1C538	1e-15	31	54	2e-15	31	54	1e-07	34	57
*Brachydanio rerio*	Q7T3C6	9e-11	32	55	1e-20	32	55	2e-12	35	57
*Xenopus laevis*	Q6DJE4	2e-20	32	55	2e-20	32	55	2e-12	35	57
*Drosophila melanogaster*	Q0E8G6	2e-19	31	54	2e-19	31	54	6e-12	34	56
*Brugia malayi*	Q8I712	1e-20	32	55	1e-20	32	55	7e-13	35	58
*Entamoeba* *dispar*	B0EHD3	e-114	86	89	e-113	96	98	4e-67	96	98
*Dictyostelium discoideum*	Q55E68	2e-26	35	61	2e-26	35	61	7e-18	37	64
*Plasmodium falciparum*	Q8I0V9	5e-16	31	51	6e-16	31	50	3e-09	30	53
*Trypanosoma brucei*	Q57WN8	7e-13	27	48	8e-13	27	48	4e-10	35	63
*Leishmania major*	Q4Q9G2	1e-11	28	47	1e-11	28	47	3e-10	36	62
*Arabidopsis thaliana*	Q8GXS3	8e-25	35	59	9e-25	35	59	4e-16	37	63
*Zea mays*	E7DDV4	3e-23	32	61	3e-23	32	61	2e-15	35	65

a UniProt Knowledgebase (UniProtKB) database; Identity (I) and similarity (S) values are in percentage

Molecular characterization of the three predicted *E. histolytica* polypeptides showed that they have most characteristics found in CFIm25 proteins. Sequence alignment predicted that both C4M2T1 and Q7YXC1 present the conserved K residue at position 45 and 26, respectively, whose acetylation modulates the interaction of CFIm25 with PAP in human [46]; they also have the conserved Y residue at position 62 and 43, respectively, that is phosphorylated in the human protein [47] (Figure S1A). In addition, bioinformatics analysis indicated that they possess the regions that have been shown to be important for RNA binding and PAP/PAB binding in the human CFIm25 protein [24] as well as the Nudix (nucleoside diphosphate linked to another moiety X) domain that has been first identified in Mut T protein from *E. coli* [48] (Figure 1B). Multiple alignment of the Nudix box sequence (consensus: GX _5_EX _7_REUXEEXGU, where U is a hydrophobic residue and X is any residue) from predicted *E. histolytica* proteins and other Nudix-containing proteins, including CFIm25 from various organisms, evidenced that C4M2T1 protein conserves the Nudix box architecture although they lack three of the four E residues that are important for catalytic function and metal binding in most Nudix-containing proteins [49]. In contrast, the B1N3I5 protein lacks the amino terminal half of the protein, including these functional residues.

In order to describe the inferred evolutionary relationships among CFIm25 proteins from several organisms including the three predicted *E. histolytica* proteins, we have undertaken a phylogenetic analysis of metazoan, plants and some protist orthologues. The full-length amino acid sequences aligned by ClustalW were used to build the corresponding tree by the Neighbor-Joining method (Figure S1B). Rooted tree topology revealed the presence of four clades. The first clade grouped all metazoan orthologues, including mammals as *H. sapiens* (O43809) and *Mus musculus* (Q9CQF3), other vertebrates as *Gallus gallus* (E1C538) and *Brachydanio rerio* (Q7T3C6), the amphibious *Xenopus laevis* (Q6DJE4), the insect *Drosophila melanogaster* (Q0E8G6) and the nematode *Brugia malayi* (Q81712). The next group included only three proteins, two plant sister proteins (Q8GXS3 and E7DDV4) with the Q55E68 protein from *Dictyostelium discoideum*, the soil-living amoeba known as slime mold, which is on the border between unicellular and multicellular, and from whom it has been suggested that it evolutionarily diverged after the plant/animal split, but before the divergence of the fungi [50,51]. The following clade grouped a pair of sister proteins from the human pathogens *Trypanosoma brucei* (Q57WN8) and *Leishmania major* (Q4Q9G2). Finally, the last clade enclosed proteins from *Plasmodium falciparum* and *Entamoeba* *dispar* , together with the three predicted *E. histolytica* proteins.

The predicted secondary structure of the largest putative EhCFIm25 (C4MT1) revealed the presence of 10 β-sheets and six α-helices. Particularly, 3D modeling showed that the conserved Nudix domain involves two mixed β-sheets flanked by two helices (α2 and α3) that form an α/β/α sandwich. The Nudix box folds into a loop-α helix-loop structure containing helix α2 (138-160 residues) and the preceding loop. The N-terminal region includes an extended loop structure (1-43 residues) followed by two short β-strands and a long α-helix (44-99 residues). The C-terminal region (205-250 residues) consists in two helices (α4 and α5) and a short β-strand (β10) (Figure 1B). The stability of the predicted 3D structure of the *E. histolytica* protein was validated by the Ramachandran plot (data not shown). Its similarity with the crystal structure of the human CFIm25 was confirmed by the RMSD value of 5.57 Å obtained from overlapping of both structures. Similar results were obtained for the Q7YZC1 protein (data not shown).

### The putative EhCFIm25 is localized in both nuclear and cytoplasmic compartments of *E. histolytica* trophozoites

**Figure 2:**
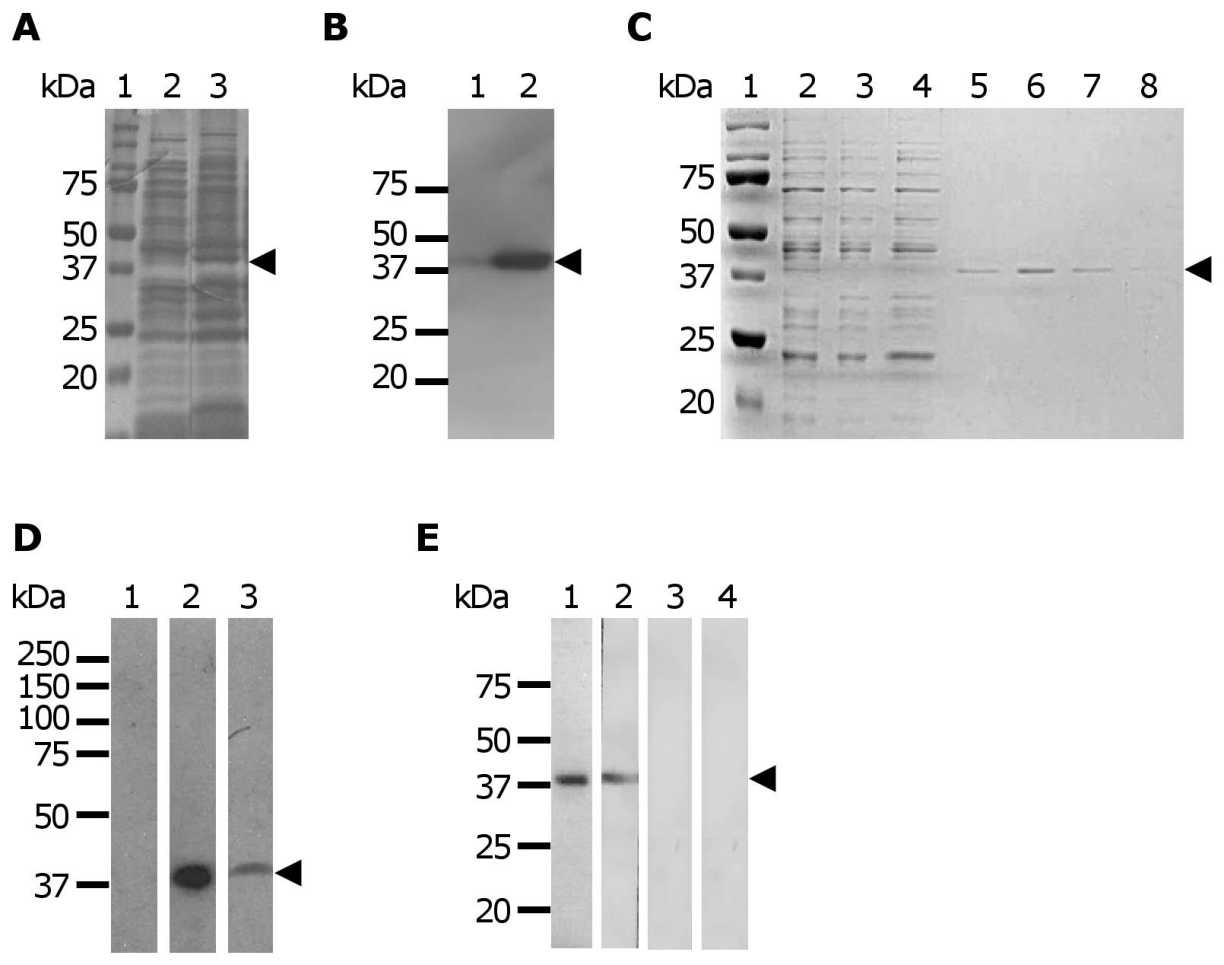
Expression of the recombinant EhCFIm25. A) Expression of the 6x-His-labeled EhCFIm25 protein. Bacteria *E. coli* were transformed with pRSET*-EhCFIm25* plasmid and protein expression was induced by the addition of 1 mM IPTG for 3 h. Proteins extracts (30 µg) were separated through 10% SDS-PAGE and gels were stained with Coomassie blue. Lane 1, molecular weight; lane 2, non-induced bacterial extract; lane 3, IPTG-induced bacterial extract. B) Immunodetection of rEhCFIm25 polypeptide by Western blot assays using anti-6x His tag antibodies. Lane 1, non-induced bacterial extract (30 μg); lane 2, IPTG-induced bacterial extract (30 μg). C) Purification of the 6x-His-labeled EhCFIm25 protein through affinity chromatography using a Ni-NTA column. Lane 1, molecular weight; lane 2, IPTG-induced bacterial extract; lane 3, unbound proteins; lane 4, wash using 150 mM imidazole; lanes 5-8, elution with 250 mM imidazole. D) Immunodetection of rEhCFIm25 polypeptide by Western blot assays using specific rabbit antibodies anti-EhCFm25Im. Lane 1, non-induced bacterial extract; lane 2, IPTG-induced bacterial extract; lane 3, IPTG-induced bacterial extract and anti-6x His tag antibodies used as control. E) Immunodetection of purified rEhCFIm25 polypeptide by Western blot assays. Lane 1, anti-6x His tag antibodies; lane 2, specific rabbit antibodies anti-EhCFIm25; lane 3, pre-immune serum; lane 4, control without primary antibody. Arrowhead, EhCFIm25.

In order to initiate the study of the putative EhCFIm25 protein in *E. histolytica*, the *EhCFIm25* gene initially identified [35] was PCR amplified using specific oligonucleotides and cloned in frame into the pRSET A plasmid. Restriction assays and DNA sequencing confirmed that the cloned sequence (768 pb) corresponds to the largest protein (C4M2T1) that has all the conserved functional characteristics. The recombinant EhCFIm25 with a 6x-His tag at the N-terminus was expressed in *E. coli* BL21 (DE3) plysS strain and observed as a 38 kDa band in gels (Figure 2A, lane 3) which was slightly higher than the predicted molecular weight (34 kDa). The same band was recognized by monoclonal anti-6x-His tag antibodies in Western blot assays (Figure 2B, lane 2). Then, rEhCFIm25 protein was purified by affinity chromatography under native conditions as shown in Figure 2C (lanes 5-8) and immunodetected by monoclonal anti-6x-His tag antibodies in Western blot assays (Figure 2E, lane 1). The purified rEhCFIm25 protein was used to generate rabbit polyclonal anti-EhCFIm25 antibodies that recognized the same 38 kDa band than monoclonal anti-6x-His tag antibodies in protein extracts from IPTG-induced *E. coli* (Figure 2D, lanes 2 and 3), confirming the specificity of the anti-EhCFIm25 antibodies. These antibodies also immunodetected the purified rEhCFIm25 (Figure 2E, lane 2), whereas the preimmune serum, used as negative control, did not give any signal (lane 3).

**Figure 3:**
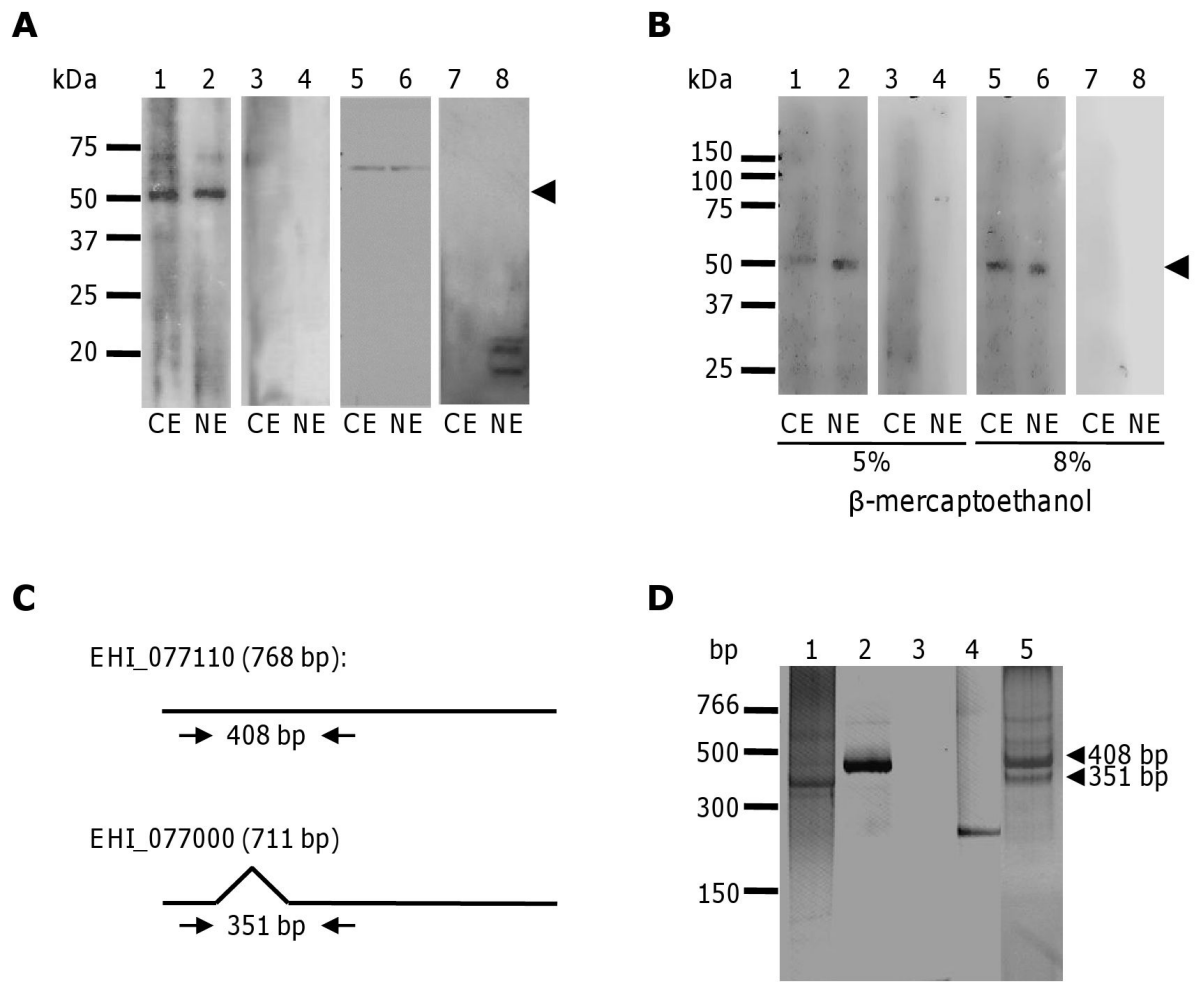
Expression of the putative EhCFIm25 in *E. histolytica* trophozoites. A and B) Western blot assays. A) Cytoplasmic (lanes 1, 3, 5 and 7) and nuclear (lanes 2, 4, 6 and 8) extracts of *E. histolytica* trophozoites were separated through 10% SDS-PAGE, electrotransferred to a nitrocellulose membrane and incubated with antibodies. Lanes 1 and 2, anti-EhCFIm25 antibodies; lanes 3 and 4, pre-immune serum; lanes 5 and 6, anti-EhPAP antibodies; lanes 7 and 8, anti-EhPC4 antibodies. B) Cytoplasmic (lanes 1, 3, 5 and 7) and nuclear (lanes 2, 4, 6 and 8) extracts were treated with 5% β-mercaptoethanol (lanes 1 to 4) or 8% β-mercaptoethanol (lanes 5 to 8) and separated through 10% SDS-PAGE in the presence of 8 M urea, and electrotransferred to a nitrocellulose membrane before being incubated with antibodies. Lanes 1, 2, 5 and 6: anti-EhCFIm25 antibodies; lanes 3, 4, 7 and 8: pre-immune serum. Arrowhead, endogenous EhCFIm25. C) Primers to evidence mRNA expression of genes at locus EHI_077110 (up) and EHI_077000 bottom) in RT-PCR assays. D) RT-PCR assays. *EhCFIm25* transcript was PCR amplified using cDNA synthesized from total RNA and products were analyzed through ethidium bromide stained polyacrylamide gels. Lane 1, molecular size markers; lane 2, cDNA; lane 3, control using genomic DNA from *E. histolytica*; lane 4, control using oligonucleotides for *actin* gene; lane 5, control using pRSET*-EhCFIm25*; lane 6, control without cDNA.

To evaluate the subcellular localization of the endogenous EhCFIm25 in *E. histolytica*, we performed Western blot assays using anti-EhCFIm25 antibodies with NE and CE from trophozoites. A signal was detected in NE as expected for a protein involved in pre-mRNA 3´ end processing; the same band was also immunodetected in CE (Figure 3A, lanes 1 and 2). No signal was detected using pre-immune serum (lanes 3 and 4). The use of antibodies against EhPAP that has been immunodetected in both NE and CE [36], confirmed protein integrity (lanes 5 and 6), while the use of antibodies against the nuclear EhPC4 [42] validated cell fractionation of both extracts (lanes 7 and 8). Intriguingly, anti-EhCFIm25 antibodies revealed a 55 kDa band whereas the predicted molecular weight for the polypeptides referred to as C4M2T1, Q7YZC1 and B1N3I5 is 30.4, 28.1 and 15.2 kDa, respectively. Therefore, we next performed Western blot assays in higher denaturing conditions in order to study whether this 55 kDa band could correspond to a protein complex that cannot be disrupted in standard SDS-PAGE conditions (Figure 3B). The same 55 kDa band was immunodetected in both NE and CE in the presence of 8 M urea and a higher concentration of β-mercaptoethanol. It was also detected when NE and CE were treated with DTT (data not shown).

To further investigate the expression of putative *EhCFIm25* genes, we performed RT-PCR experiments using primers that were designed to amplify products of different size for transcripts corresponding to locus EHI_077110 and EHI_077000 (Figure 3C and D). Results evidenced the presence of two bands with the expected molecular weight (351 bp and 408 bp), which confirmed that both genes are transcribed, although gene at locus EHI_077110 seems to be weakly transcribed in our experimental conditions (lane 1). Control assay using genomic DNA showed the presence of the same bands (lane 5), which correspond to both genes described above. As expected, the 408 bp was obtained from pRSET vector (lane 2). The other controls included RT-PCR using actin oligonucleotides (lane 3) and RT (-) (lane 4).

### The putative EhCFIm25 exhibits RNA binding activity *in vitro*

**Figure 4:**
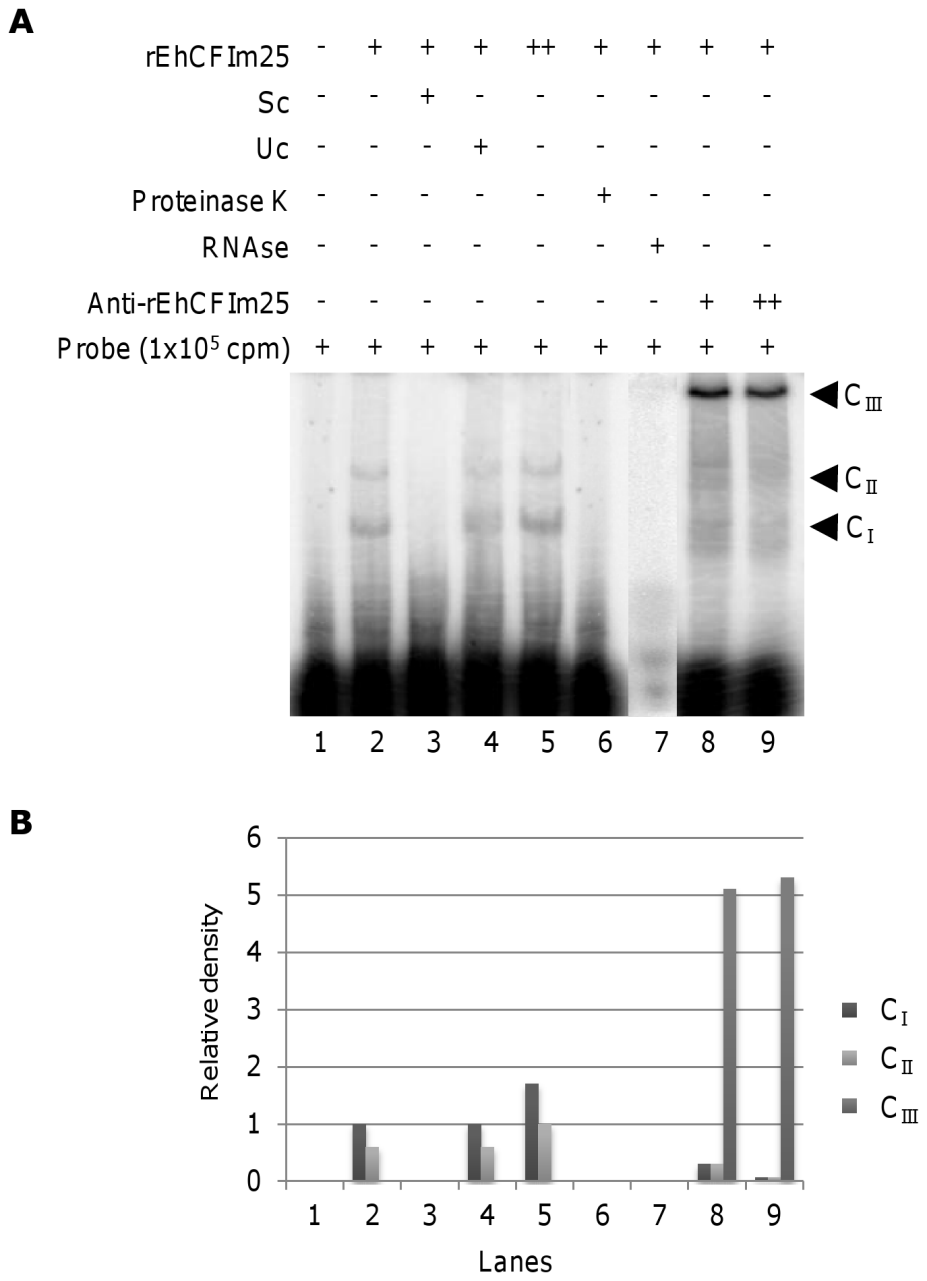
RNA binding activity of rEhCFIm25. A) REMSA. rEhCFIm25 was incubated with [α-^32^P] UTP-labeled PSIII^156^ RNA probe (5×10^5^ cpm) at 4 °C for 15 min. Complexes were resolved through 6% non-denaturing PAGE and detected in a Phosphor Imager apparatus. Lane 1, free probe; lane 2, RNA probe plus 5 nM rEhCFIm25; lane 3, as in lane 2 plus specific competitor (Sc) (350-fold molar excess of unlabeled probe); lane 4, as in lane 2 plus unspecific competitor (Uc) (350-fold molar excess of unlabeled tRNA); lane 5, RNA probe plus 10 nM rEhCFIm25; lane 6, as in lane 2 plus proteinase K; lane 7, as in lane 2 plus RNAse; lanes 8 and 9, as in lane 2 plus antibody anti-EhCFIm25. Arrowhead, RNA-protein complex. B) Densitometry analysis of complexes detected in A. Pixels corresponding to C_I_ in lane 2 was taken as 100% and used to normalize data.

Binding to RNA substrate is required for CFIm25 functions. To test whether the putative EhCFIm25 can bind RNA, we performed RNA Electrophoretic Mobility Shift Assays (REMSA) using the 256 nt [α-^32^P] UTP-labeled PSIII^156^ RNA fragment that contains the last 100 nt of the open reading frame and 156 nt of the 3′ UTR of the *EhPgp5* gene [43] as a probe (Figure 4). We decided to use the 3′ UTR of the *EhPgp5* gene because it contains all the potential pre-mRNA 3´ UTR *cis*-regulatory sequences described in *E. histolytica*, including PAS, poly(A) site, U-rich tracts, and A-rich elements [52]. Results showed that purified rEhCFIm25 was able to bind RNA on its own. The presence of two RNA-complexes (C_I_ and C_II_) could be due to the interaction of alternative populations of rEhCFIm25 proteins to RNA probe, producing complexes with distinct electrophoretic mobility (lane 2). RNA-protein complexes were specifically competed by a 350-fold molar excess of the same unlabeled transcript, while they were maintained in the presence of tRNA, used as unspecific competitor (lanes 3 and 4), indicating the preference of rEhCFIm25 for pre-mRNA 3´ end. The abundance of both RNA-protein complexes was about 1.5-fold increased in the presence of a higher amount of rEhCFIm25 (lane 5). Moreover, both complexes disappeared in control experiments performed in the presence of proteinase K (lane 6) and RNase (lane 7), showing the presence of both RNA and protein components in the complexes. Finally, the presence of specific antibodies against EhCFIm25 led to the formation of a third complex (C_III_) with a slower migration, together with the disappearance of the fastest complexes C_I_ and C_II_ (lanes 8 and 9), which confirms the presence of rEhCFIm25 in the RNA-protein complexes.

### The putative EhCFIm25 interacts with the poly(A) polymerase EhPAP

**Figure 5:**
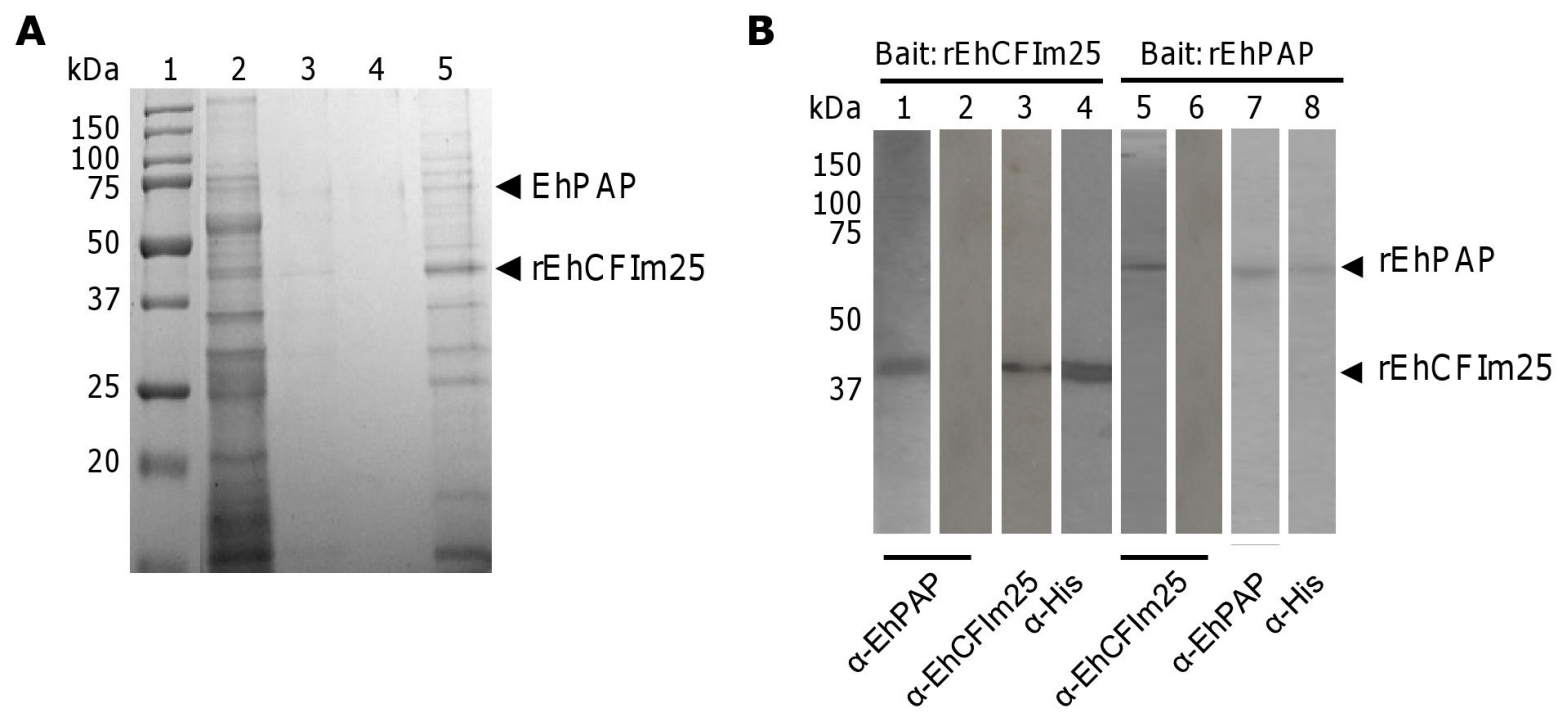
*In vitro* protein-protein interaction assays. A) Pull-Down assay. The rEhCFIm25 immobilized on Ni^2+^-NTA column was incubated with NE. After washing, proteins were eluted, separated though 10% SDS-PAGE and stained with Coomassie Brilliant blue. Lane 1, Molecular weight markers; lane 2, proteins not retained on the column; lanes 3 and 4, washing fraction; lane 5, elution fraction. B) Far-Western assay. Purified rEhCFIm25 (lanes 1 to 4) and rEhPAP (lanes 5 to 8) were subjected to 10% SDS-PAGE and electrotransferred to a nitrocellulose membrane that was incubated with rEhPAP (lane 1) or rEhCFIm25 (lane 5). Proteins were immunodetected by specific antibody anti-EhPAP (lanes 1, 2 and 7) or anti-EhCFIm25 (lanes 3, 5 and 6) and revealed by the ECL Plus Western blotting system (Amersham). Anti-His antibody was used as control (lanes 4 and 8).

It has been described that the human CFIm25 interact with other polyadenylation factors, as well as with proteins from other pre-mRNA processing machineries. In order to evaluate the interactions of the putative EhCFIm25 with other nuclear proteins, we performed a Pull-Down assay using rEhCFIm25 immobilized on a Ni^2+^-NTA column and *E. histolytica* NE that were previously passed through a Ni^2+^-NTA column to eliminate non-specific interactions, as prey (Figure 5A). Nuclear proteins that were not retained on the rEhCFIm25-Ni^2+^-NTA column were removed (lane 2) and unspecific interactions were eliminated by extensive washes (lanes 3 and 4) before bound proteins were eluted (lane 5). After SDS-PAGE analysis and Coomassie blue staining, results revealed that the elution fraction contains a strong band at the molecular weight corresponding to rEhCFIm25. We also observed the presence of other bands with different molecular weights (< 20 kDa up to ~150 kDa). These bands were excised and proteins were identified by LC/ESI-MS/MS tandem mass spectrometry analysis and NCBI database search. As expected, results confirmed that the 37 kDa band correspond to rEhCFIm25. Interestingly, they also indicated that the 60 kDa band was the poly(A) polymerase EhPAP.

Because it has been reported that the 25 kDa subunit of the Cleavage Factor Im interacts with the poly(A) polymerase in human [24,32], we decided to corroborate the interaction of EhCFIm25 with EhPAP in *E. histolytica* by Far-Western assays (Figure 5B). First, purified rEhCFIm25 was immobilized in the nitrocellulose membrane and rEhPAP protein was used as a prey. Specific anti-EhPAP antibody recognized the 38 kDa band (lane 1) that was also detected in control Western blot assay using rEhCFIm25 (lane 3) and anti-His (lane 4) antibodies. In contrast, no signal was obtained with anti-EhPAP antibody (lane 2). When purified rEhPAP was immobilized in the membrane and rEhCFIm25 protein was used as a prey, specific anti-EhCFIm25 antibody recognized a protein that corresponds to the molecular weight of rEhPAP (lane 5) as it is evidenced in control Western blot assay using rEhPAP (lane 7) and anti-His (lane 8) antibodies. In contrast, no signal was obtained with anti-CFIm25 antibody (lane 6).

## Discussion

Little is known about pre-mRNA 3´ end cleavage and polyadenylation pathways and machineries in protozoan parasites. Recently, the analysis of the *E. histolytica* genome allowed us to identify the putative RNA sequences and proteins involved in mRNA 3´ end formation in this organism [35,52]. In this work, we focused on the putative 25 kDa subunit of the Cleavage Factor Im, EhCFIm25, which is the unique subunit of the Cleavage Factor Im in *E. histolytica*.

Bioinformatics analysis showed that the predicted *E. histolytica* C4M2T1 protein conserves the characteristic features of human CFIm25, which strongly suggests that it could be the homologue of the 25 kDa subunit described in human. Notably, it has the Nudix domain, which has been described first in *E. coli* [48] and then in other proteins, including the human CFIm25, although it diverges from the Nudix consensus: specifically, three of the four glutamate residues of the consensus sequence have been replaced by K154, K157 and K158, respectively, and the last G residue of the motif was substituted by serine, an hydrophilic residue. However, the putative EhCFIm25 still harbors the classical Nudix fold consisting of an α/β/α sandwich [53]. *E. coli* NudD and human NUDT9H also lack several glutamate residues [54] without affecting their folding [27,30,55] neither their RNA binding activity [24]. Notably, human CFIm25, which lacks two of the conserved glutamate residues that are important for catalytic function and metal binding [48,49], does not possess a hydrolytic activity, although it could still bind nucleotides [27]. Thus, it is possible that the K154, K157 and K158 residues in the putative EhCFIm25 Nudix motif are important for structural stabilization of the Nudix helix and the preceding β-strand, as it has been shown for human CFIm25 [27,55]. The observation that 25 kDa subunits of CFIm are unconventional Nudix proteins is supported by the fact that they are not able to cleave RNA. Indeed, biochemical and structural studies evidenced that CPSF-73 that directly binds to the poly(A) site and possesses a Ca^2+^-dependent ribonuclease activity, is the endoribonuclease responsible for the pre-mRNA cleavage [22]. The C-terminal and N-terminal regions of the putative EhCFIm25 also conserves the folding described for the human CFIm25 protein, including the extended loop structure at the N-end that is important for RNA binding [25]. These features are also present in the predicted Q7YC1 protein whose gene was identified in the recently actualized *E. histolytica* genome database, which suggested that both predicted proteins could be the homologues of the human CFIm25. Interestingly, RT-PCR assays indicated that both genes are transcribed in our experimental conditions, which is in agreement with data from microarrays analysis reported in *E. histolytica* database. In contrast, the smallest protein (B1N3I5) is probably not functional, if it is expressed, since it lacks the amino terminal half of the sequence.

The largest protein (C4M2T1) was expressed in *E. coli* and purified in native conditions to initiate its characterization. The immunolocalization of the endogenous protein in the nucleus, where RNA synthesis and pre-mRNA 3´ end processing take place, is in good agreement with the biological function of a factor involved in nuclear processes. However, its presence in cytoplasm is more unexpected. In *E. histolytica*, EhPAP has also been immunodetected in both nuclear and cytoplasm fraction [36]. Intriguingly, the endogenous protein was immunodetected as a 55 kDa band by specific antibodies anti-EhCFIm25, even in highly denaturing conditions. The human CFIm is a dimer of 25 kDa subunits with an apparent molecular weight of ^~^53 kDa, interacting with the larger subunits of 59 and 68 kDa [27,30]. Because 55 kDa is about the double of the molecular weight predicted for the putative EhCFIm25, this band may correspond to a stable dimer of EhCFIm25, whose resistance to dissociation could be due to the existence of interwoven structures and strong residues association. A similar feature has been previously described for the 112 kDa adhesin of *E. histolytica* [56] and the catalase HPII from *E. coli* that exhibited an enhanced stability [57]. Nuclear extracts contain many other proteins, including other pre-mRNA 3´ end processing proteins that could also be interacting with the putative endogenous EhCFIm25 to form the 55 kDa stable complex. If such interactions occur, they should require the presence of other parasite proteins since the 55 kDa band was not detected in IPTG-induced *E. coli* extracts neither during the purification protocol. Another possibility is that the predicted EhCFIm25 has an aberrant electrophoretic mobility as it has been reported for other *E. histolytica* proteins [58]. Further experiments using immunoassays, chromatographic methods, spectrophotometric analysis and circular dicroism assays, should help to confirm the precise nature of this 55 kDa polypeptide. The absence of smaller bands confirmed that the smallest polypeptide is not expressed in the experimental conditions used here.

In a standard REMSA, rEhCFIm25 was able to bind a 3´ UTR RNA sequence in the absence of any other mRNA 3´ end processing factors. Similarly, the human CFIm can bind RNA on its own; neither CstF nor PAP had an influence on the binding of CFIm on pre-mRNA 3´ end, although the addition of CPSF led to the formation of a more slowly migrating complex [25]. The putative EhCFIm25 does not have a classical RNA binding domain, but its N-terminal region includes the same extended loop structure including the conserved K45 residue that allows human CFIm25 to bind RNA [25,46]. The results obtained from gel retardation assays suggested that EhCFIm25 binds to the *EhPgp5* pre-mRNA 3´ UTR with a certain degree of specificity. This RNA sequence has the *cis*-regulatory motifs described in *E. histolytica* [52], but it does not contain the UGUAN (N=A>U≥C/G) sequence which is recognized by the human CFIm25 [19,20,23,28,29], which suggests that EhCFIm25 may bind to another RNA motif. Further assays currently in progress will help to map the region on the pre-mRNA substrate that is recognized the 25 kDa subunit in *E. histolytica*.

The putative EhCFIm25 presents the PAP binding domain described in the human CFIm25 protein [24] with the conserved K45 residue [46]. Pull-Down and Far-Western experiments evidenced that EhCFIm25 was able to interact with EhPAP *in vitro*. PAP is a key enzyme in the pre-mRNA 3´ end processing machinery: it is recruited to the RNA molecule since the beginning of the transcription [59] and is responsible for the synthesis of poly(A) tail at the 3´ end of mRNA [21]. An interaction between mammalian CFIm25 and PAP has been previously described from yeast two-hybrid screens and Pull-Down assays [24,32,60]. The observation that EhCFIm25 interacts with EhPAP suggests that it may be introduced into the processing complex since the early steps of the cleavage/polyadenylation reaction through this protein–protein interaction, as it has been described in Mammals [24].

## Conclusions

In summary, our results showed that *E. histolytica* only has the 25 kDa subunit of Clevage Factor Im, which is able to interact with RNA and the poly(A) polymerase EhPAP, another member of the pre-mRNA 3´ end processing machinery in this human pathogen.

## Supporting Information

Figure S1 Computational analysis of predicted EhCFIm25. A) Comparative analysis of amino acid sequences from the three predicted CFIm25 polypeptides in *E. histolytica* genome. Numbers at the right are relative to the initial methionine in each protein. Functional K and Y residues are underlined. Asterisk, identical aa. B) Phylogenetic relationships among CFIm25 proteins from several organisms including three *E. histolytica* putative CFIm25.The evolutionary history was inferred using the Neighbor-Joining method and 1000 replicates in order to obtain the bootstrap consensus tree. Branches corresponding to partitions reproduced in less than 50% bootstrap replicates were collapsed. The percentage of replicate trees in which the associated taxa clustered together in the bootstrap test is shown next to the branches. The UniProt Knowledge Base accession number for each protein is indicated for all organisms.
(TIF)
