# Supplementary figures and images for: The 25 kDa Subunit of Cleavage Factor Im Is a RNA-Binding Protein That Interacts with the Poly(A) Polymerase in Entamoeba histolytica
Source: PLoS One. 2013 Jun 28;8(6):e67977. doi: 10.1371/journal.pone.0067977 (PMC3695940; doi:10.1371/journal.pone.0067977)

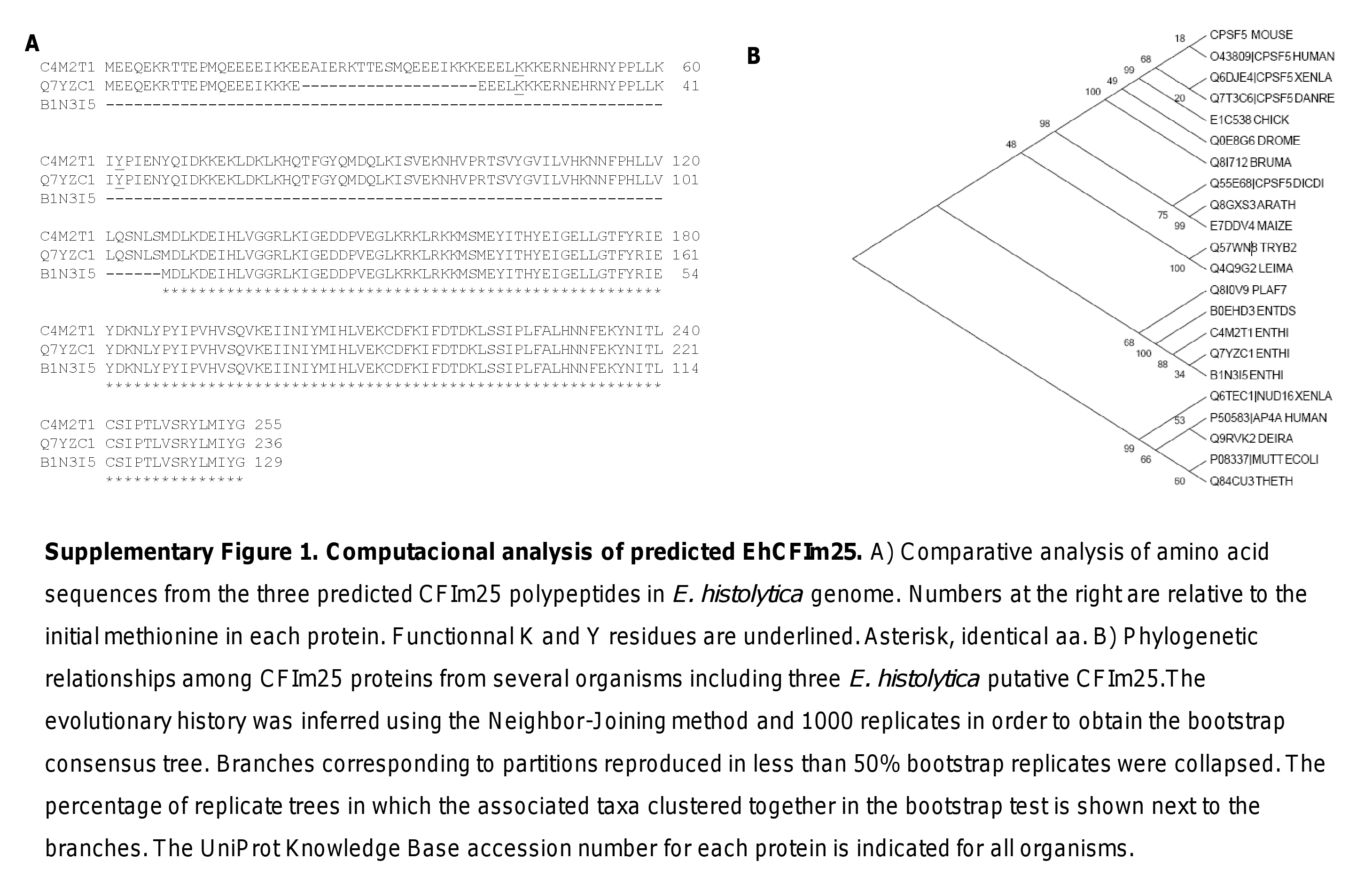

Supplement: Figure S1 — A) Comparative analysis of amino acid sequences from the three predicted CFIm25 polypeptides in E. histolytica genome. Numbers at the right are relative to the initial methionine in each protein. Functional K and Y residues are underlined. Asterisk, identical aa. B) Phylogenetic relationships among CFIm25 proteins from several organisms including three E. histolytica putative CFIm25.The evolutionary history was inferred using the Neighbor-Joining method and 1000 replicates in order to obtain the bootstrap consensus tree. Branches corresponding to partitions reproduced in less than 50% bootstrap replicates were collapsed. The percentage of replicate trees in which the associated taxa clustered together in the bootstrap test is shown next to the branches. The UniProt Knowledge Base accession number for each protein is indicated for all organisms. (TIF) [file pone.0067977.s001.tif]
